# Mixed Th1/Th2/Th17 Responses Induced by Plant Oil Adjuvant-Based *B. bronchiseptica* Vaccine in Mice, with Mechanisms Unraveled by RNA-Seq, 16S rRNA and Metabolomics

**DOI:** 10.3390/vaccines12101182

**Published:** 2024-10-17

**Authors:** Xuemei Cui, Qiuju Xiang, Yee Huang, Quanan Ji, Zizhe Hu, Tuanyuan Shi, Guolian Bao, Yan Liu

**Affiliations:** 1 Institute of Animal Husbandry and Veterinary Science, Zhejiang Academy of Agricultural Sciences, Hangzhou 310021, China; maycui@zju.edu.cn (X.C.); xxqiu@stu.zafu.edu.cn (Q.X.); huangyee@zaas.ac.cn (Y.H.); jiquanan@zaas.ac.cn (Q.J.); zizhehu@zaas.ac.cn (Z.H.); tuanyuanshi@zaas.ac.cn (T.S.); 2 College of Animal Science and Technology·College of Veterinary Medicine, Zhejiang A&F University, Hangzhou 311300, China

**Keywords:** E515 adjuvant, immunity, *Bordetella bronchiseptica*, mechanisms

## Abstract

Background/Objectives: The current *Bordetella bronchiseptica* (Bb) vaccine, when adjuvanted with alum, does not elicit adequate robust cellular immunity or effective antibody defense against Bb attacks. Unfortunately, antibiotic treatment generally represents an ineffective strategy due to the development of resistance against a broad range of antibiotics. Methods: The present study was designed to investigate the immune response, protective capabilities and underlying mechanisms of a plant oil-based adjuvant E515 formulated with inactivated Bb antigen as a potential vaccine candidate against *Bordetella bronchiseptica*. Results: Immunization studies revealed that a combination of SO, VE and GS (E515) exhibited a good synergistic adjuvant effect. The E515 adjuvanted Bb vaccine was proven to be highly efficacious and induced a mixed Th1/Th2/Th17 immune response in mice, leading to a significant increase in Bb-specific IgG, IgG1 and IgG2a antibodies, proliferative lymphocyte responses and cytokine levels (by lymphocytes and serum) and effectively induced responses by CD4^+^ TE, TM cells and B cells. The E515 adjuvant significantly enhanced the immune protection provided by the Bb vaccine in a mice model, as indicated by a reduced bacterial burden in the lungs. Multi-omics sequencing analysis revealed that E515 functions as an adjuvant by modulating critical pathways, including cytokine–cytokine receptor interaction, the IL-17 signaling pathway and the chemokine signaling pathway. This modulation also included interactions with beneficial species of bacteria including *Alistipes*, *Odoribacter* and *Colidextribacter*, as well as energy and lipid-related metabolites, thus highlighting its role as an immunomodulatory agent. Conclusion: Collectively, our results demonstrate the huge potential of E515-Bb vaccine candidates, thus highlighting the vegetable oil original adjuvant E515 as a promising agent for the development of new veterinary vaccines.

## 1. Introduction

*Bordetella bronchiseptica* (Bb) belongs to the *Alcaligenaceae* family *Bordetella* genus, and is a Gram-negative bacterium that plays a role in a diversity of infectious respiratory diseases in mammals [1]. Bb has a broad host range, including pigs, dogs, rabbits, rodents, and occasionally, humans. Infected animals can exhibit mild or severe clinical symptoms, including porcine atrophic rhinitis, canine infectious respiratory syndrome and rabbit rhinitis. Bb disease spreads rapidly by contact with infected animals, or by contaminated fomites and can even be transmitted between different susceptible animals. Furthermore, Bb is often co-infected with other pathogens, leading to several secondary herd diseases [2]. Consequently, Bb can cause significant economic losses in livestock farms on an annual basis.

Antibiotics are widely used to treat Bb diseases at present. However, with the increasing resistance of Bb to antibiotics, the efficacy of these antibiotics is gradually decreasing [2,3], coupled with the prevalence of Bb disease in mammals and the increased number of cases involving human infections. Bb infection has become a serious public health issue [4]; consequently, there is an urgent need to develop new prevention and treatment strategies. Vaccines are powerful weapons that have substantially reduced the burden of Bb diseases, especially in dogs and pigs [5,6]. Nevertheless, a specific vaccine aimed at preventing Bb infections in rabbits has yet to be developed. Consequently, there is a pressing necessity to devise an effective vaccine that can control Bb infections in rabbits and diminish the risk of potential transmission from rabbits to humans. Adjuvants are a very important component of vaccines because they enhance the strength and longevity of vaccine-induced immune responses and can also influence the specific type of immune responses [7]. Aluminum hydroxide (alum) and mineral oil are the most extensively used adjuvants for veterinary vaccines. Alum adjuvant-based vaccines usually activate humoral but weak cellular immune responses. However, these types of vaccines also induce side effects such as skin irritation at the site of inoculation, a severe inflammatory response, or allergic reactions [8,9]. Mineral oil-based adjuvants, such as Montanide^TM^ series ISA 61 VG, 201 VG and 206 VG, are mainly dependent on imports. However, these adjuvants also induce side effects post-injection and their poor metabolism restricts their wider application; in particular, these vaccines are not suitable for animals with fur or animals that are used for meat [10]. Our preliminary research revealed that the administration of ISA 61 VG, 201 VG and 206 VG as adjuvants for Bb vaccines led to severe side effects in rabbits after immunization by subcutaneous injection.

In contrast with the adjuvants described above, plant original oils are renewable, cheap, edible and safe. The potential advantages of using vegetable oil as an adjuvant are vast, encompassing environmental friendliness, improved product performance and stability, enhanced consumer appeal, potential health benefits and reduced environmental impact. These advantages make vegetable oil a compelling choice for various applications where an adjuvant is required. A sunflower seed oil-based adjuvant was previously verified to be much safer than a mineral oil adjuvant and white oil [11]. Interestingly, our research team found that a soybean oil-based adjuvant consisting of α-tocopherol (VE) and ginseng saponins (GS) named E515, had a good safety profile following immunization in mice [12], Hu sheep [13] and pigs. In addition to its safety profile, E515 can significantly enhance the humoral and cellular immune responses induced by the foot-and-mouth disease (FMD) vaccine [12]. Recent studies have shown that a combination of humoral Th1/Th17 cellular responses are essential for protection against Bordetella [1,14]. In this study, we specifically analyzed the T cell-Th1/Th2/Th17/Treg and B cell immune responses induced by the E515-adjuvanted Bb vaccine in a mouse model from multiple perspectives and used integrated multi-omics analysis to explore the potential mechanisms of action.

## 2. Materials and Methods

### 2.1. Animals

Female ICR mice (age, 6–8 weeks of age; weight, 20−24 g; number, 108 total) were provided by the Shanghai Animal Center Co., Ltd., (Shanghai, China). The mice were housed in cages maintained at a stable temperature (22 ± 3 °C) and humidity (50 ± 10%) with a 12/12 h light/dark cycle and had free access to food and water. The study was performed after the mice were allowed to acclimate for 7 days. All the animal experiments were approved by the ZAAS Ethics Committee (Reference: 2021ZAASLA17).

### 2.2. Adjuvant and Vaccine Preparation

Soybean oil was obtained from Zhejiang Tian Yu Shan Medicinal Co., Ltd. (Zhejiang, China), α-tocopherol (VE) (purity ≥ 96%) was purchased from Sigma (Saint Louis, MI, USA), and standardized GS was obtained from Hongjiu Ginseng Industry Co., Ltd. (Jilin, China). Alum adjuvant was acquired from Maximmune Pharmaceutical Technology Co., Ltd. (Chengdu, China). Bb (FX strain) was cultured according to a previously reported method [15] and sterilized with 0.4% formalin for 24 h. The inactivated bacterial protein was combined with E515 or alum in a 1:2 (*v*/*v*) ratio and the adjuvanted vaccines contained 3 × 10^9^ CFU/mL of antigen. To prepare the vaccines, the E515-adjuvanted Bb vaccine mixture was emulsified with an IKA T10 basic emulsification machine (Staufen, Germany) at a shear rate of 14,500 rpm for 2 min to form an emulsion (oil-in-water, O/W) [13]. The alum-adjuvanted Bb vaccine was prepared according to the manufacturer’s protocol: alum was added to the Bb antigen with constant mixing for 20–30 min to form a stable formula. Alum was usually mixed with antigen at least one day before animal inoculation, thus ensuring that the Bb antigen was effectively adsorbed by the alum (alum-Bb vaccine).

### 2.3. Animal Immunization

**Figure 1:**
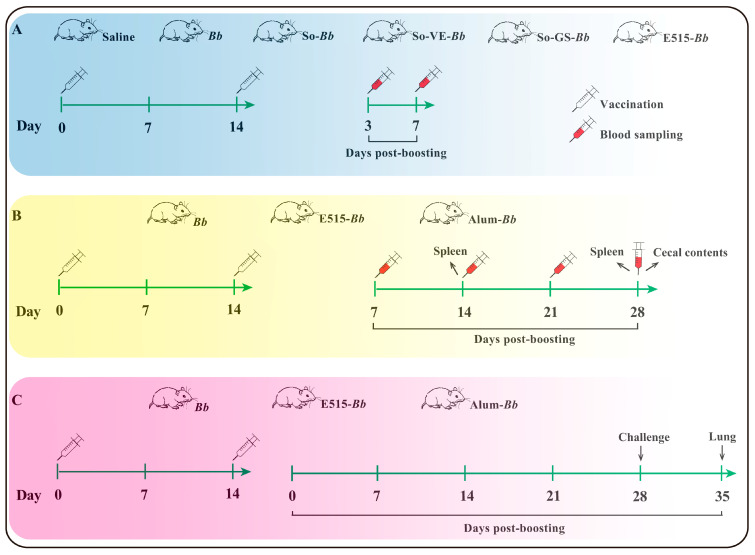
Experimental design. (**A**) Synergistic effects of E515 adjuvant on Bb vaccine. Mice (n = 6/group) were immunized intramuscularly (i.m.) on days 0 and 14. Blood samples were collected 3 and 7 days after boost to measure Bb-specific IgG. (**B**) Immune effects of the E515-Bb vaccine. Mice (n = 15/group) were i.m. Blood samples were collected 7, 14, 21 and 28 days after boosting to measure Bb-specific IgG, IgG1 and IgG2a. On day 14, spleens were collected to measure lymphocyte proliferation, relative mRNA expression, T/B cell differentiation and cytokine production (spleen and serum). On day 28, spleens and cecal contents were collected for transcriptomic, 16S rRNA sequencing and untargeted metabolomics analyses. (**C**) Protection effect of E515-Bb vaccine. Mice (n = 9/group) were immunized i.m. on day 0. On day 28 post-boosting, mice were challenged with live Bb (5.2 × 10^9^ CFU/mice); seven days later lungs were collected for bacterial burdens quantified.

There are three experiments in this study:

Experiment 1: ICR mice were randomly allocated into six groups (n = 6 per group) and were intramuscularly (i.m.) inoculated twice with 0.2 mL of saline, a Bb antigen and SO-Bb, SO-GS-Bb, SO-VE-Bb or E515-Bb at a 2-week interval, respectively. Serum samples were collected 3 and 7 days after boosting to test for Bb-specific IgG (Figure 1A, Table 1);

Experiment 2: ICR mice were randomly assigned into three groups (n = 15 per group) and were i.m. injected twice with 0.2 mL of Bb antigen, E515-Bb or alum-Bb at a 2-week interval. Serum samples were collected 7, 14, 21 and 28 days after boosting to detect Bb-specific IgG, IgG1 and IgG2 and cytokines. Two weeks after booster immunization, the spleens of the mice in each group were aseptically collected for cell proliferation assays, flow cytometry testing, the detection of transcription factor expression and cytokine production. Four weeks after booster immunization, the spleens from the mice in each group were collected for transcriptomic analysis; cecal contents were also collected for gut microbiome and untargeted metabolomics analyses (Figure 1B, Table 2);

Experiment 3: ICR mice were randomly assigned into three groups (n = 9 per group) and were injected (i.m.) twice with 0.2 mL of Bb antigen, E515-Bb or alum-Bb at a 2-week interval. Then, the mice were challenged by an intraperitoneal injection of live Bb (5.2 × 10^9^ CFU/mice) 28 days after boosting. Seven days after the attack, the bacterial loads in the lungs of the mice from each group were evaluated (Figure 1C, Table 3).

### 2.4. Bb-Specific IgG and Isotype Analysis

The levels of Bb-specific IgG and IgG isotypes were detected by indirect ELISA. The inactivated Bb protein was used to coat a 96-well microtiter plate (1 μg/mL, 100 μL/well) which was incubated at 4 °C overnight. The plates were then washed 3–5 times with PBST solution (PBS+ 0.05% Tween-20), followed by blocking with 5% skimmed milk at 37 °C for 2 h. After washing, 100 µL of diluted serum samples (1:200) was added to each well and the plate was incubated at 37 °C for 1 h. Next, 100 µL of HRP-conjugated goat anti-mouse IgG (1:20,000) or IgG1 (1:5000) and IgG2a (1:5000) was added to each well and incubated at 37 °C for 1 h. Next, after washing, 100 μL of tetramethyl–benzidine reagent was added to each well and incubated at 37 °C for 8–15 min in the dark. Then, 50 µL of stop solution (2 M H_2_SO_4_) was added to each well and the optical density (OD) of the plate was read at 450 nm with a microplate reader (Thermo-Multiskan, Shanghai, China).

### 2.5. Cytokine Arrays

The serum samples were analyzed by a glass-based and sandwich-based antibody microarray that allowed the quantitative analysis of 18 different cytokines (Mouse Cytokine Array GS1, RayBiotech, Inc., Guangzhou, China). Each cytokine was analyzed in a quadruplicate per array. In brief, 100 µL of serum were added to each well, incubated overnight at 4 °C and then extensively washed. A biotin-labeled detection antibody was then added and incubated for 2 h followed by the application of AlexaFluor 555-conjugated streptavidin at room temperature for 1 h. The slides were analyzed by an InnoScan 300 Microarray Scanner with 532 nm excitation and 635 nm emission. We obtained raw data (images) from the scanner and integrated spot intensities (tab-delimited .txt file) by Mapix v.7.3.1 software. Data was visualized by Q-Analyzer Software (v24.5.29) and all raw data were log2-converted for statistical analysis.

### 2.6. Lymphocyte Proliferation

The spleens were isolated under aseptic conditions and gently passed through a fine steel mesh to obtain a splenocyte suspension. Following centrifugation at 1500 rpm for 5 min in 4 °C, the supernatant was discarded. Erythrocytes were lysed with red blood cell lysis buffer (Solarbio Life Science, Beijing, China) for 5 min. Then, the buffer was removed by centrifugation and the cells were re-suspended in complete RPMI 1640 medium (RPMI supplemented with 10% inactivated fetal bovine serum, 100 IU/mL of penicillin and 100 μg/mL of streptomycin). Then, 100 µL of splenocytes (5 × 10^6^ cells/mL) were added to 96-well plates and stimulated by 100 µL of Con A (10 µg/mL), LPS (10 µg/mL), Bb antigen (20 µg/mL) or RPMI 1640 medium, respectively. After incubation for 48 h at 37 °C in a 5% CO2 environment, 10 µL of CCK-8 reagent (APExBIO, Houston, TX, USA) was added and the cells were incubated for an additional 2–4 h. Finally, the absorbance at 450 nm was measured with a microplate reader. The stimulation index (SI) was determined using the following formula: OD values of mitogen-stimulated wells/OD values of non-stimulated wells [16].

### 2.7. Cytokine Production by Splenocytes

The splenocytes (5 × 10^6^ cells/ML) were stimulated with inactivated Bb antigen (20 µg/mL) for 72 h (37 °C, 5% CO_2_). After centrifugation (1500 rpm, 5 min), the supernatant was collected for the measurement of cytokines (IFN-γ, IL-6, TGF-β1 and IL-17) by commercial ELISA kits (Multi-Sciences Biotech, Hangzhou, China) in accordance with the manufacturer’s instructions.

### 2.8. Flow Cytometry

For the T lymphocyte subsets, the splenocytes (5 × 10^6^ cells/ML) were stained with a mixture of fluorochrome-conjugated anti-mouse antibodies to CD3-FITC, CD4-PerCP-Cy5.5 and CD8-APC. For memory/effector T lymphocytes, the splenocytes were stained with CD3-Alexa Fluor, CD4-PE-Cy7, CD8-PE, CD62L-APC and CD44-FITC. For the B cells, the splenocytes were stained with CD3-Alexa Fluor, CD19-APC, CD38-PerCP-Cy5.5, CD138-PE and FAS-FITC. All the antibodies were acquired from Multi-Sciences (Hangzhou, China). The stained splenocytes were incubated at room temperature for 30 min in the dark. After centrifugation at 1500 rpm for 8 min, the supernatant was discarded; then, the cells were re-suspended in PBS and were analyzed with a FACS Canto™ flow cytometer (BD Biosciences, Franklin Lake, NJ, USA). The data was analyzed by FLOW JO software (version 10.0).

### 2.9. Real-Time Quantitative Polymerase Chain Reaction

The splenocytes (1 × 10^7^ cells/ML) were stimulated with inactivated Bb antigen (20 µg/mL) for 24 h (37 °C, 5% CO_2_). Total RNA was extracted with a SPARK-easy Cell RNA Kit and cDNA was generated by a reverse transcription SPARK-script RT Plus Kit (with gDNA eraser) (Sparkjade Biotechnology Co., Ltd., Qingdao, China). Quantitative PCR was performed using a 2 × SYBR Green qPCR Mix Kit (Sparkjade, Qingdao, China) on an ABI Prism 7500 system (Biosystems, Shanghai, China). The PCR cycling conditions were as follows: 94 °C for 3 min, and 40 cycles of amplification at 94 °C for 10 s and 60 °C for 30 s. The data for relative quantification was analyzed by the comparative CT method (2^−ΔΔCT^) [17]. All the sampling processes were carried out within a sterile clean bench. The transcription factors GATA-3, T-bet and ROR-γt were detected and GAPDH was employed as reference gene [18]. All the primers were synthesized by Sangon Biotech (Shanghai, China) (Table S1).

### 2.10. Transcriptomic Analysis

Total RNA was extracted with a Trizol reagent kit (Invitrogen, Carlsbad, CA, USA) in accordance with the manufacturer’s protocol. The RNA quality was assessed on an Agilent 2100 Bioanalyzer (Agilent Technologies, Palo Alto, CA, USA) and checked by RNase-free agarose gel electrophoresis. Then, mRNA was enriched by Oligo (dT) and reverse transcribed into cDNA with a NEBNext Ultra RNA Library Prep Kit for Illumina (New England Biolabs, Ipswich, MA, USA). The purified double-stranded cDNA fragments were end-repaired and ligated to Illumina sequencing adapters. The ligation reaction was purified with AMPure XP Beads (1.0X). The resulting cDNA library was sequenced with an Illumina Novaseq6000 by Gene Denovo Biotechnology Co. (Guangzhou, China). Differential RNA expression analysis between the two different groups was performed by DESeq2 software (v3.9). The genes with a false discovery rate (FDR) < 0.05 and an absolute fold change ≥ 2 were considered as differentially expressed genes (DEGs). All the DEGs were subjected to Gene Ontology (GO) and Kyoto Encyclopedia of Genes and Genomes (KEGG) enrichment analyses. The sequencing data have been deposited into the China National Center for Bioinformation (CNCB) database under the accession number CRA014305.

### 2.11. 16S rRNA Sequencing

Microbial DNA was extracted and purified with a HiPure DNA stool Kit (Magen, Guangzhou, China) and an AxyPrep DNA Gel Extraction Kit (Axygen Biosciences, Union City, CA, USA). The V3-V4 regions of the 16S rRNA genes were amplified by the primers B341F and B806R (B341F: CCTACGGGNGGCWGCAG, B806R: GGACTACHVGGGTATCTAAT). The PCR products were purified by AMPure XP Beads (Axygen, CA, USA), quantified with a Real-Time PCR System (ABI, Oakland, CA, USA) and sequenced on an Illumina NovaSeq platform. Based on ≥97% sequence similarity, the sequences were clustered into operational taxonomic units (OTUs) by UPARSE (version 9.2.64) software. Representative OTU sequences were classified and annotated into organisms by a naive Bayesian model using RDP (Ribosomal Database Project) based on the SILVA database. An alpha diversity analysis, including ACE, Chao1, Shannon and Simpson indices, was calculated using QIIME software (version 1.9.1). A beta diversity analysis, including a PCoA (principal coordinates analysis) and the NMDS (non-metric multi-dimensional scaling) of weighted unifrac distances, was generated in the R project Vegan package (version 2.5.3) and plotted in the R project ggplot2 package (version 2.2.1). Differentially abundant taxa were identified by a linear discriminant analysis (LDA) and linear discriminant analysis effect size (LEfSe) analyses. The statistical analysis involved a Welch’s *t*-test, Wilcoxon’s rank test, Tukey’s HSD test, the Kruskal–Wallis H test, Adonis (also known as Permanova) and a Anosim test; these tests were performed in the R project Vegan package [14] (version 2.5.3). The sequencing data have been deposited into the CNCB database under the accession number CRA014349.

### 2.12. Metabolomics Analysis

The cecal content was determined by UHPLC-MS/MS- (high-performance liquid chromatography-mass spectrometry) based untargeted metabolomics. The samples (100 mg) were mixed with 80% methanol by vortexing, incubated on ice (5 min) and centrifuged (15,000× *g*, 15 min). The supernatant was diluted to a final concentration containing 53% methanol by LC-MS grade water and collected by centrifugation (15,000× *g*, 20 min) into another tube. Finally, the filtrate was injected into the LC-MS/MS system for analysis. PLS-DA was applied to discriminate the different variables between groups using the R package (http://www.r-project.org/). A variable importance in the projection (VIP) score/(O)PLS model was conducted to rank the metabolites that best distinguished between the two groups. The threshold for VIP was set to 1. In addition, the T-test was also used for univariate analysis to identify differential metabolites. Those with a p value (T test) < 0.05 and a VIP ≥ 1 were considered as differential metabolites between the two groups. KEGG pathways meeting at FDR ≤ 0.05 were defined as significantly enriched pathways in differential metabolites. The sequencing data have been deposited into the CNCB database under the accession number PRJCA022627.

### 2.13. Validation of DEGs by RT-PCR

Twelve of the DEGs were selected to verify the transcriptomic sequencing results by RT–PCR. The primers for DEGs were synthesized by Sangon Biotech (Table S1) and RT-PCR was performed as described in Section 2.9.

### 2.14. Bacterial Loads

The mice were sacrificed by cervical dislocation and their lungs were collected and homogenized. The obtained tissue suspensions were serially diluted (1:10, 1:100 and 1:1000) and 100 µL of dilution was inoculated in duplicate on trypticase soy agar (TSA) (Oxoid, Basingstoke, UK) plates. Bacterial colonies were counted after culturing for 18–24 h at 37 °C.

### 2.15. Statistical Analysis

GraphPad Prism 8.0 software (USA) was used for data analysis. A Shapiro–Wilk Normality test was employed to check data distribution. Multiple comparisons were conducted by two-way ANOVA followed by Tukey’s multiple comparisons test. The results are expressed as the mean ± SE and *p* < 0.05 was considered statistically significant. The correlation between gut microbiota communities and metabolites was conducted by Pearson’s correlation coefficients and *p* < 0.05 was considered statistically significant.

## 3. Results

### 3.1. E515-Bb Immunization Enhanced Bb-Specific Humoral Immune Responses

**Figure 2:**
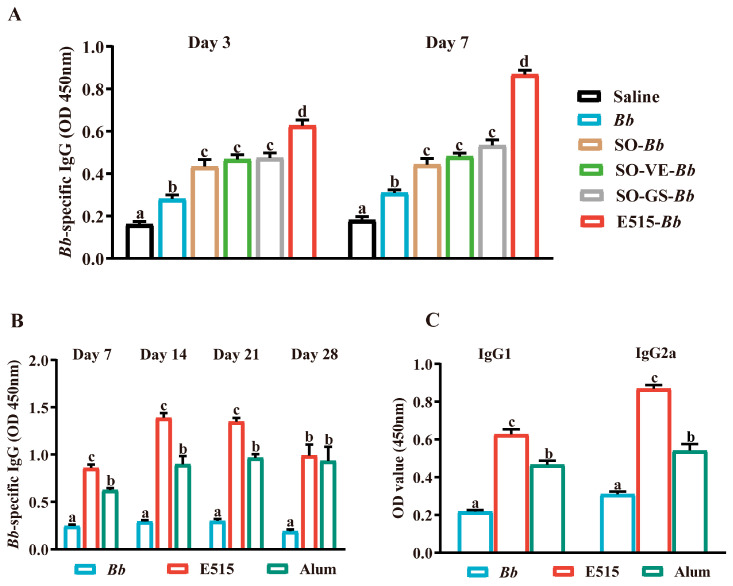
Vaccine-induced humoral immune response (Mice, n = 6/group). (**A**) Blood samples were collected 3 and 7 days after boost to measure Bb-specific IgG. (**B**) Serum samples were collected 7, 14, 21 and 28 days after boosting to measure Bb-specific IgG. (**C**) IgG1 and IgG2a were measured 14 days after the boost. Data shown as mean ± SE. Bars with different letters are statistically different (*p* < 0.05).

First, we investigated the E515 adjuvant effect and the synergistic effects of VE and GS in SO on the Bb-specific antibody response in mice. Analysis showed that, 3 and 7 days after second immunization, compared to the Bb antigen group, the SO-Bb, SO-VE or SO-GS group induced higher levels of Bb-specific IgG antibody in mice (*p* < 0.05). The highest level of Bb-specific IgG was detected in the E515-Bb group (*p* < 0.05) (Figure 2A). As indicated, 7, 14, 21 and 28 days after boosting, both E515 and alum augmented the IgG response elicited by the Bb vaccine. A significant increase in the IgG antibody in the first three weeks post-boost was observed in the E515 (E515-Bb) immunized group (*p* < 0.05). However, from 28 days after the second injection, there was no difference between the two adjuvanted Bb vaccine groups (Figure 2B). A surge in IgG isotype (IgG1 and IgG2a) responses were also observed in the E515 group 14 days after the booster; this was significantly higher than the Bb antigen and alum (alum-Bb) groups (*p* < 0.05) (Figure 2C). These results suggest that E515-Bb is a more effective Bb vaccine candidate for inducing a Bb-specific humoral reaction.

### 3.2. E515-Bb Immunization Heightened Bb-Specific Cellular Immune Responses

**Figure 3:**
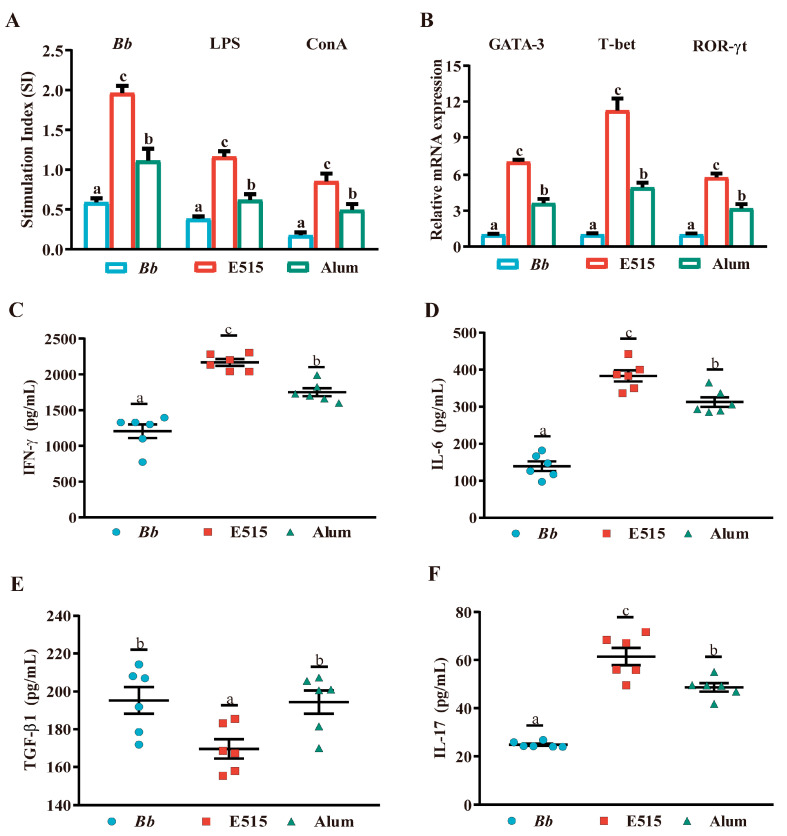
Vaccine-induced cellular immune response (Mice, n = 6/group). Splenocytes were isolated from spleens 14 days after the boost. (**A**) SI splenocytes were stimulated by Con A (10 µg/mL), LPS (10 µg/mL) or Bb antigen (20 µg/mL) for 48 h. (**B**) The expression of GATA-3, T-bet and ROR-γt mRNA splenocytes were stimulated by the Bb antigen (20 µg/mL) for 24 h. (**C**–**F**) The concentration of the IFN-γ, IL-6, TGF-β1 and IL-17 splenocytes were stimulated by the Bb antigen (20 µg/mL) for 72 h. Data shown as mean ± SE. Bars with different letters are statistically different (*p* < 0.05).

The CCK-8 method was applied to detect the proliferation of splenic lymphocytes in different groups after stimulation with a Con A, LPS or Bb antigen. The analysis showed that the lymphocyte SI proportion in the E515 and alum groups were significantly increased. Compared to the alum group, E515 produced a higher proliferative ability when the lymphocytes were stimulated with a Con A, LPS or Bb antigen (*p* < 0.05) (Figure 3A). Figure 3B shows that the mice in the E515 group had significantly higher expression levels of GATA-3, T-bet and ROR-γt mRNA than the mice in the other two groups (*p* < 0.05). This result was consistent with the lymphocyte proliferation findings. The analysis revealed significantly higher levels of IFN-γ, IL-6 and IL-17 cytokines in the mice immunized in the alum group when compared to the Bb antigen group (*p* < 0.05). However, no significant difference was detected in the production of TGF-β1 when compared between the groups. Compared with those in the alum group, the mice in the E515 group produced higher levels of IFN-γ IL-6 and IL-17, and lower levels of TGF-β1 (*p* < 0.05) (Figure 3C–F). These results suggested that E515-Bb is an effective vaccine candidate for inducing and balancing a wide type of Th1/Th2/Th17/Treg cellular immune response.

### 3.3. E515-Bb Immunization Modulated Cytokine Production in Serum

**Figure 4:**
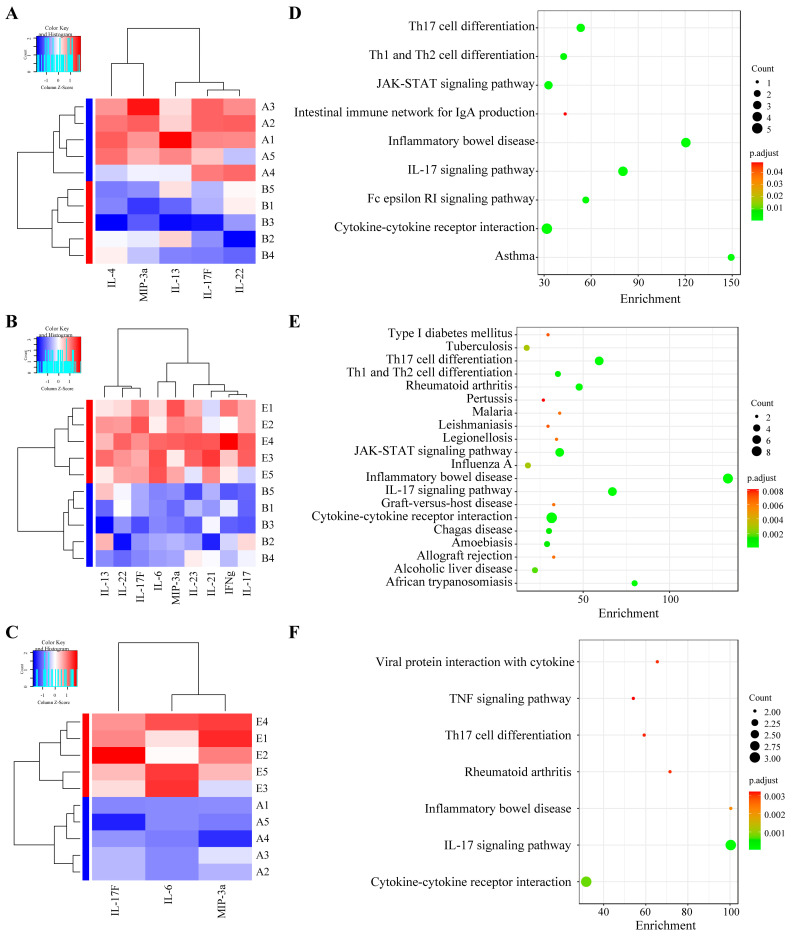
Cytokine production in serum (Mice, n = 5/group). Serum samples were collected 14 days after the boost and detected by a cytokine array. (**A**) Heat map of hierarchical clustering of cytokines in Bb vs. alum. (**B**) Heat map of hierarchical clustering of cytokines in Bb vs. E515. (**C**) Heat map of hierarchical clustering of cytokines in alum vs. E515. (**D**) KEGG pathway enrichment of cytokines in Bb vs. alum. (**E**) KEGG pathway enrichment of cytokines in Bb vs. E515. (**F**) KEGG pathway enrichment of cytokines in alum vs. E515. Red color on the heatmap indicates cytokines are upregulated and blue indicates cytokines are downregulated. Dot size on the KEGG pathway represents the number of cytokines; the color represents the *p* value.

A mouse cytokine antibody array was adopted to identify 18 cytokines in serum samples. An analysis showed that two weeks after booster immunization, four cytokines and one chemokine in the alum group (IL-4, IL-13, IL-22, IL-17F and MIP-3α) (Figure 4A) and eight cytokines and one chemokine in the E515 group (IL-13, IL-22, IL-17F, IL-6, IL-23, IL-21, IFN-γ, IL-17 and MIP-3α) (Figure 4B) were significantly increased when compared to the Bb antigen group (*p* < 0.05). Furthermore, significant elevations of IL-17F, IL-6 and MIP-3α in the E515 group were observed when compared to the alum group (*p* < 0.05) (Figure 4C). The KEGG pathway results implied that, compared to Bb antigen group, Th17/Th1/Th2 cell differentiation, cytokine–cytokine receptor interaction, the Th17 signaling pathway and the JAK-STAT signaling pathway, were significantly activated in the two adjuvanted vaccine groups (Figure 4D,E). The TNF signaling pathway, Th17 cell differentiation, the Th17 signaling pathway and the cytokine–cytokine receptor interaction signaling pathway were clearly enriched in the E515 vs. alum comparison (Figure 4F). These results suggested that E515-Bb immunization induced the production of Th1/Th2/Th17 hybrid cytokines.

### 3.4. E515-Bb Immunization Regulated T-Cell Subsets, TE and TM Cells and B Cells in the Spleen

**Figure 5:**
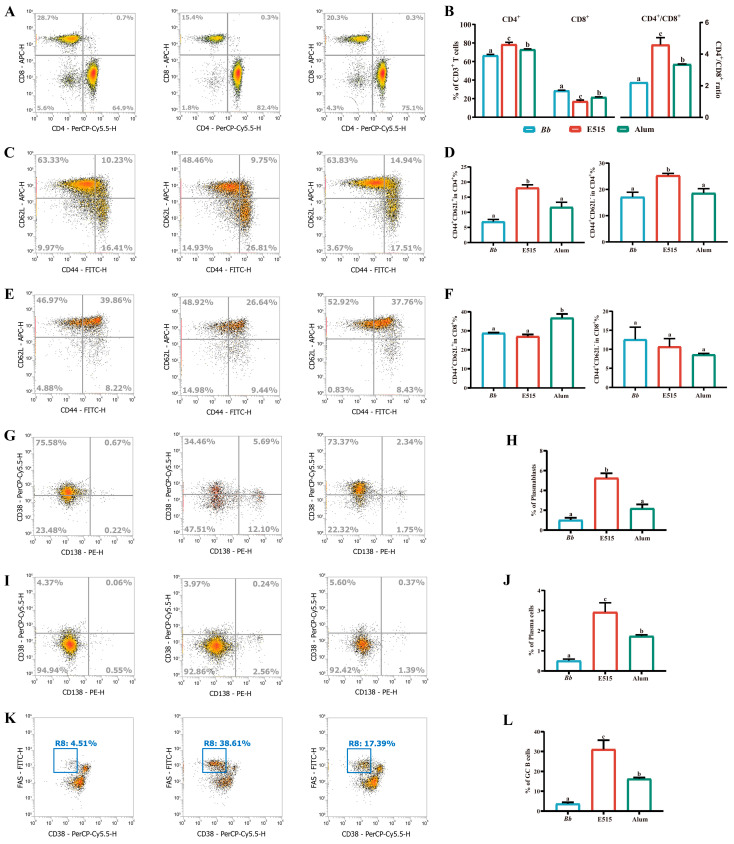
Bb-specific T cell and B cell responses in the spleen (Mice, n = 5/group). Splenocytes were isolated from the spleens 14 days after the boost, and analyzed by flow cytometry. (**A**,**B**) Frequencies and quantification of the CD4^+^, CD8^+^ T cells and CD4^+^/CD8^+^ ratio. (**C**,**D**) Frequencies and quantification of the CD4^+^ TM (CD44^+^ CD62L^+^) and TE (CD44^+^ CD62L^−^) cells. (**E**,**F**) Frequencies and quantification of the CD8^+^ TM and TE cells. (**G**,**H**) Frequencies and quantification of the plasmablasts (CD19^+^ CD138^+^ CD38^+^). (**I**,**J**) Frequencies and quantification of the plasma cells (CD19^−^ CD138^+^ CD38^−^). (**K**,**L**) Frequencies and quantification of the GC B cells (CD19^+^ Fas^+^ CD38^−^). Data shown as mean ± SE. Bars with different letters are statistically different (*p* < 0.05).

The lymphocytes were isolated from the spleens in different groups for flow cytometric analysis. The frequency of the CD4^+^ T cells and the ratio of the CD4^+^/CD8^+^ T cells were significantly higher (E515 > alum > Bb, *p* < 0.05) and the CD8^+^ T cells were significantly lower (E515 < alum < Bb, *p* < 0.05) in the spleens of the mice after vaccination with E515 and alum than in those vaccinated with the Bb antigen (*p* < 0.05) (Figure 5A,B). In the spleens of the E515-immunized mice, the CD4^+^ TM and TE cells were significantly increased (*p* < 0.05), although no significant differences were detected in the CD8^+^ TM and TE cells when compared to alum and Bb. Alum-immunized mice exhibited a significantly higher frequency of CD8^+^ TM and TE cells (*p* < 0.05), but not CD4^+^ TM and TE cells (Figure 5C–F). The frequency of B cells in the splenocytes were as follows: E515 > alum ≥ Bb, *p* < 0.05 (Plasmablasts) (Figure 5G,H), E515 > alum > Bb, *p* < 0.05 (Plasma cells) (Figure 5I,J), E515 > alum > Bb and *p* < 0.05 (GC B cells) (Figure 5K,L). These results suggest that immunization with the E515-Bb vaccine candidate enhanced Bb-specific T cell and B cell responses in the spleen.

### 3.5. Differential Gene Expression Analysis

**Figure 6:**
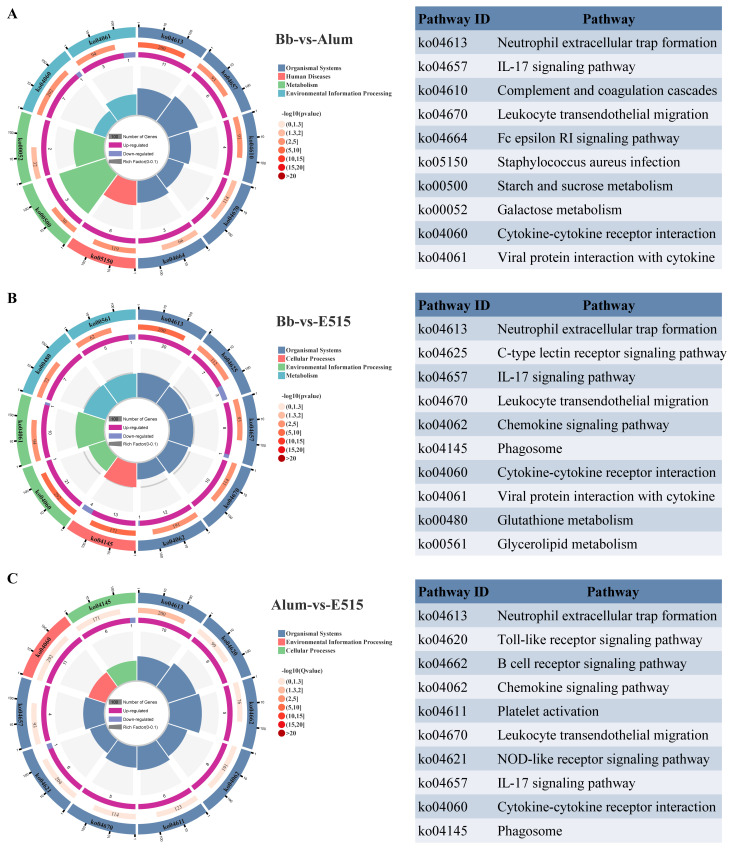
KEGG pathways analysis (n = 3 /group). Comparison of DEGs KEGG modules between Bb-vs-alum (**A**), Bb-vs-E515 (**B**) and alum-vs-E515 (**C**).

Intrigued by the results relating to humoral and cellular responses, the splenic genes expression profiles were investigated using RNA-seq analysis. A DEG analysis identified 510 genes (421 up, 89 down) with significant changes in the Bb-vs-E515. Compared to the Bb group, 161 DEGs (139 up, 22 down) were significantly changed following alum intervention. In addition, 263 genes were upregulated and 40 genes were downregulated in the alum-vs-E515 comparison (Figure S1A). In addition, 46 DEGs were shared across all the groups; 234, 21 and 110 DEGs were unique in each of the group pairs (Figure S1B). The top 30 DEGs with a maximum log2 (fc) were presented as a radar chart, including Reg, Ighv, Stfa and Cstds in Bb-vs-E515; Ighv, Ppihl, Ighe and Gm49450 in Bb-vs-alum; and Reg, Ighv and Dynlt5 in alum-vs-E515 (Figure S1C–E). Compared to the alum or Bb antigen group, the E515 adjuvanted Bb vaccine group predominantly activated immune-related GO terms associated with immune system processes, responses to stimulus, defense responses and the regulation of immune system processes (Figure S2). In the E515 or alum-adjuvanted vaccine groups, KEGG pathways such as neutrophil extracellular trap formation, cytokine–cytokine receptor interaction, the IL-17 signaling pathway and viral protein interaction with cytokine and cytokine receptors, were enriched when compared to the Bb antigen group. A few pathways were enriched for E515 but not alum, including phagosomes, the C-type lectin receptor signaling pathway, leukocyte transendothelial migration and the chemokine signaling pathway. Some pathways involved in the complement and coagulation cascades, leukocyte trans endothelial migration, the Fc epsilon RI signaling pathway and carbohydrate metabolism were activated in the alum (Figure 6).

### 3.6. Diversity Analysis of the Gut Microbiota

**Figure 7:**
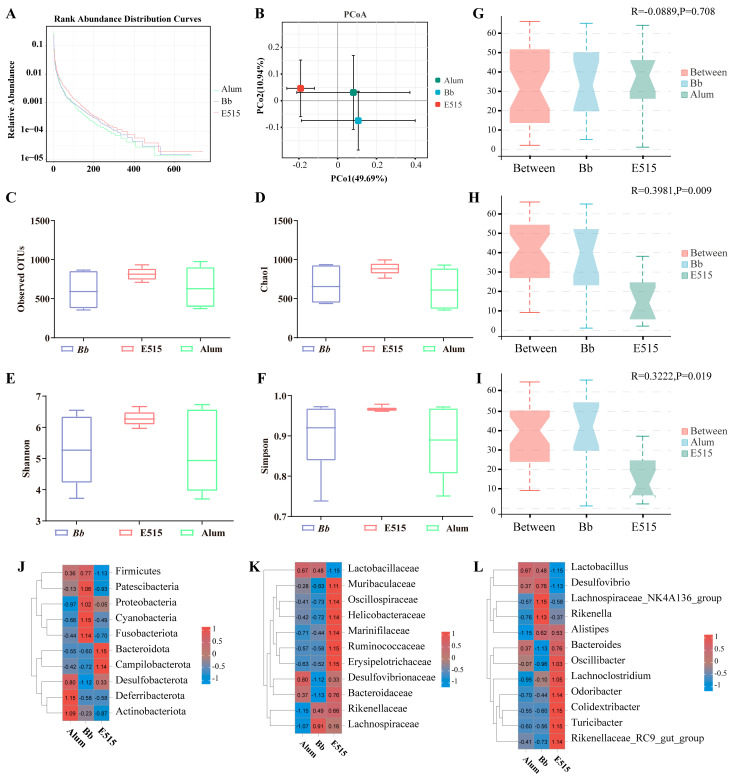
Effect of E515 on the diversity and composition of gut microbiota (n = 6/group). (**A**) Rank–abundance curve. (**B**,**G**–**I**) β-diversity was detected using PCoA (principal coordinates analysis) and ANOSIM (non-metric multi-dimensional scaling). (**C**) Observed OTUs (operational taxonomic units). (**D**–**F**) α-Diversity was detected using Chao1, Shannon and Simpson indices. (**J**–**L**) Composition of gut microbiota was analyzed at the phylum, family and genus levels.

To investigate the potential regulatory role of E515 or alum combined with an Bb antigen on gut microbiota in mice, we analyzed the cecal contents using 16 S rRNA sequencing. A rank–abundance curve was generated to explain the species abundance and evenness (Figure 7A); the most diverse mice were those in the E515 group while the greatest OTU abundance was detected in the E515 group (Figure 7C). Alpha-diversity, including the Chao1, Simpson and Shannon indices, illustrated the abundance and diversity of microorganism communities across the samples. As shown, α-diversity did not differ between the three groups (Figure 7D–F). Principal coordinates analysis (PCoA) revealed that mice in the E515 group showed no significant differences in β-diversity when compared to the other two groups (Figure 7B). This was confirmed by ANOSIM (analysis of similarities) testing (Bb-vs-E515, R = 0.40, *p* = 0.009; alum-vs-E515, R = 0.32, *p* = 0.019). The ANOSIM test also revealed significant differences in the bacterial community structure between groups (Figure 7G–I).

**Figure 8:**
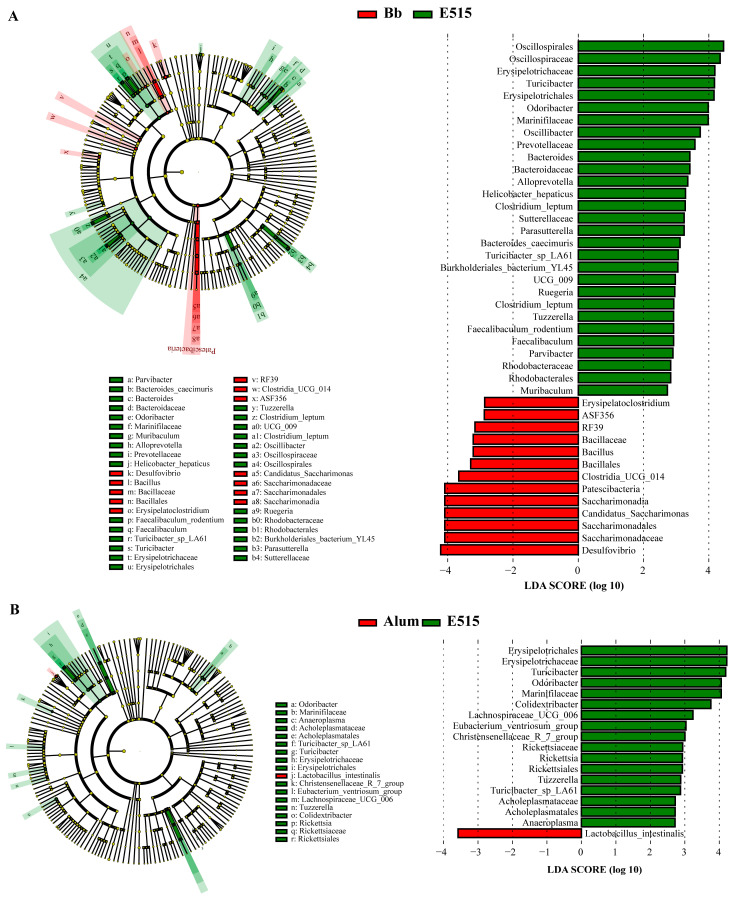
Differences in microbial abundances were identified by linear discriminant analysis (LDA, LDA score > 2) and linear discriminant analysis effect size (LEfSe) analyses. (**A**) Bb-vs-E515. (**B**) alum-vs-E515.

**Figure 9:**
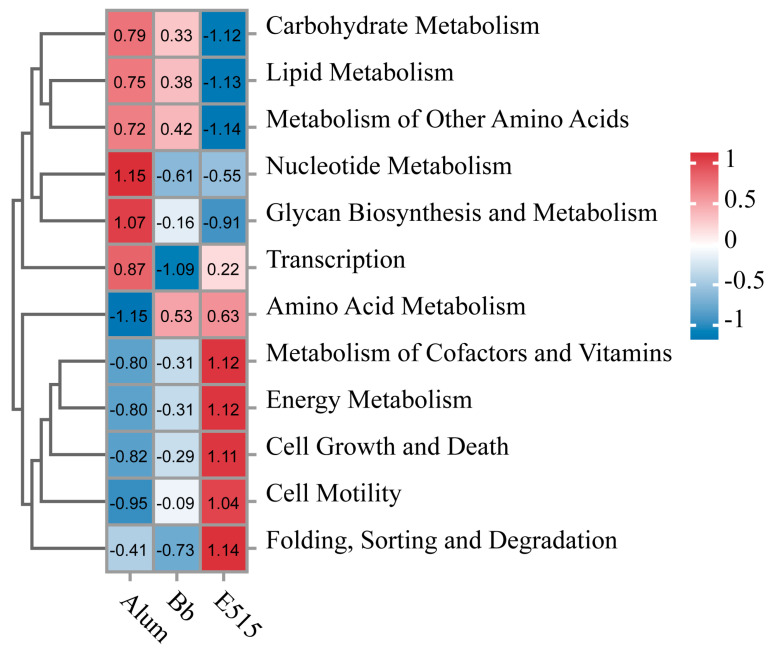
KEGG pathway analysis of differential microbes in the three groups.

To further understand the microbial dissimilarities between the groups, we evaluated the taxonomic composition of the cecal microbiota. At the level of the phylum, Firmicutes, Bacteroidetes, Desulfobacterota and Campilobacterota, were the main phyla. The mice in the E515 group showed a reduction in Firmicutes (54% in the Bb group, 56% in the alum group, 46% in the E515 group) and an increase in Bacteroidetes (29% in the Bb group, 29% in the alum group, 38% in the E515 group) and Campilobacterota (2% in the Bb group, 2% in the alum group, 4% in the E515 group) (Figure 7J) (Figure S3A). A detailed investigation of the family of Firmicutes revealed reductions in Lactobacillaceae and increases in Oscillospiraceae and Lachnospiraceae. The abundance of Bacteroidetes was represented by increases in Muribaculaceae, Rikenellaceae and Marinifilaceae in the mice after treatment with the E515-Bb vaccine (Figure 7K) (Figure S3B). At the genus level, E515 significantly reduced the abundance of Lactobacillus, Desulfovibrio and the Lachnospiraceae_NK4A136_group and promoted the abundance of Alistipes, Odoribacter and Colidextribacter (Figure 7L) (Figure S3C). LEfSe analyses were conducted (LDA score > 2) to further evaluate the differences in the bacterial composition within the different groups. Figure 8A showed that the relative abundance of 29 bacteria, including Oscillospirales, Odoribacter and Turicibacter, was significantly upregulated in the E515 group, while that of 13 bacteria, including Desulfovibrio, Saccharimonadaceae and Candidatus_Saccharimonas, was downregulated when compared to the Bb group (*p* < 0.05). The relative abundance of 17 bacteria, including Turicibacter, Erysipelotrichaceae and Odoribacter, was significantly upregulated, while the abundance of Lactobacillus_intestinalis was significantly downregulated in the E515 group when compared to the alum group (*p* < 0.05) (Figure 8B). These results were consistent with those arising from the Cladogram analysis. In the KEGG profiles, pathways such as “Metabolism of Cofactors and Vitamins”, “Energy Metabolism”, “Cell Growth and Death”, “Cell Motility”, “Translation” and “Folding, Sorting and Degradation” were the most abundant in the E515 group. Some pathways, such as “Nucleotide Metabolism” and “Glycan Biosynthesis and Metabolism” were enriched in the alum group (Figure 9).

### 3.7. Untargeted Metabolomics Analysis

**Figure 10:**
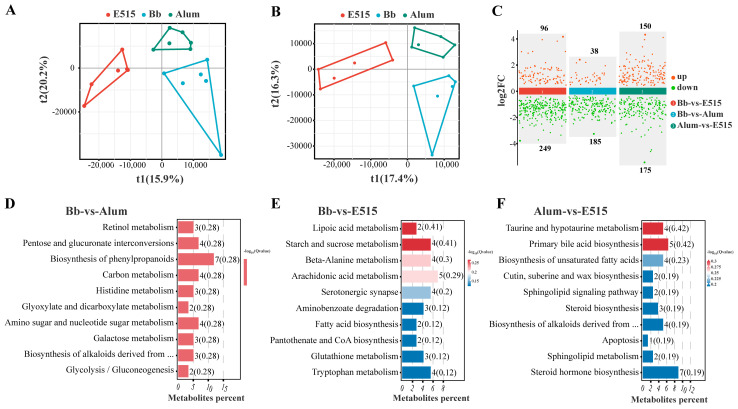
Effect of E515 on the changes of gut metabolic (n = 6 /group). (**A**) PLS-DA analysis score plots of the metabolic profiles in the negative ion mode. (**B**) PLS-DA analysis score plots of the metabolic profiles in the positive ion mode. PLS-DA and OPLS-DA analysis score plots of the metabolic profiles in the negative ion mode. Cross-validation results in the negative ion mode. (**C**) Differential expressed metabolites (DEMs) in every comparison group (VIP > 1 and *p* < 0.05). (**D**–**F**) KEGG pathway analysis of the differential metabolites in every comparison groups.

To investigate the metabolic changes in the mice treated with the two adjuvanted Bb vaccines or the Bb antigen, we performed an untargeted metabolomics analysis of the cecal content. A total of 4333 metabolites (1948 negative and 2385 positive mode) compounds were identified in the cecal metabolome. A partial least squares discriminant analysis (PLS-DA) was carried out to separate the different groups; the analysis showed that the samples from the Bb, E515 and alum groups were distinctly classified into three clusters, with significant differences between each group (Figure 10A,B). Next, we screened differential expressed metabolites (DEMs) with a VIP > 1 and *p* < 0.05. Compared to the Bb antigen group, 345 DEMs (96 up, 249 down) or 223 DEMs (38 up, 185 down) were significantly changed after the Bb antigen was combined with E515 or alum adjuvant. Furthermore, 150 DEMs were upregulated and 175 DEMs were downregulated in the alum-vs-E515 comparison (Figure 10C). The top 10 enriched pathways were listed for every two-group comparison. In the Bb-vs-E515 comparison, five out of the 10 pathways, including “Lipoic acid metabolism” (*p* = 0.006), “Starch and sucrose metabolism” (*p* = 0.008), “Beta-Alanine metabolism” (*p* = 0.015), “Arachidonic acid metabolism” (*p* = 0.021) and “Serotonergic synapse” (*p* = 0.033) were significantly activated. In the Bb-vs-alum comparison, four out of the 10 pathways were significantly enriched, including “Retinol metabolism” (*p* = 0.007), “Pentose and glucuronate interconversions” (*p* = 0.013), “Biosynthesis of phenylpropanoids” (*p* = 0.021) and “Carbon metabolism” (*p* = 0.051). In the alum-vs-E515 comparison, three out of the 10 pathways were significantly activated: “Taurine and hypotaurine metabolism” (*p* = 0.007), “Primary bile acid biosynthesis” (*p* = 0.009) and “Biosynthesis of unsaturated fatty acids” (*p* = 0.020) (Figure 10D–F).

### 3.8. Correlation Analysis between Gut Microbiota and Differential Metabolites

**Figure 11:**
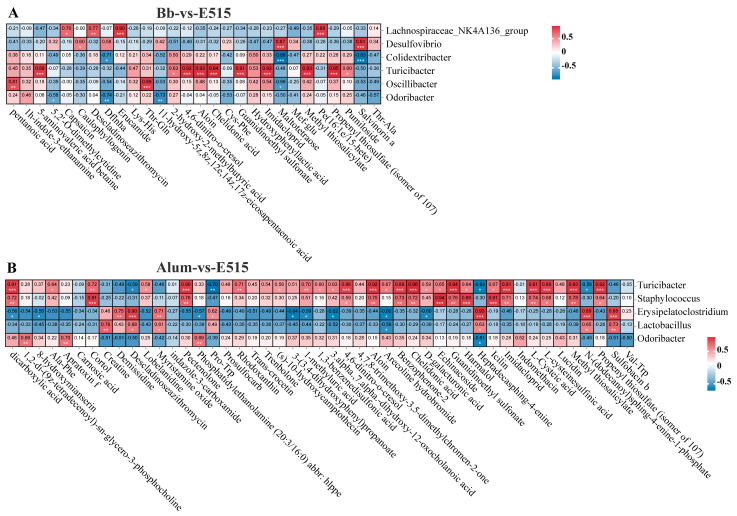
Pearson’s correlation analysis among metabolites and microbes. (**A**) Bb-vs-E515. (**B**) Alum-vs-E515. Positive correlation marked with red color, negative correlation marked with green color, * *p* < 0.05, ** *p* < 0.01, *** *p* < 0.001.

Pearson’s correlation coefficient (|R| > 0.58, *p* < 0.05) was employed to evaluate the relationship between the gut microbiota and metabolites. In the Bb-vs-E515 comparison (Figure 11A), beneficial microbes *Colidextribacter* and *Odoribacter* were negatively correlated with three (Dflnha, Maltotetraose, Salvinorin a) and two (Dflnha, eicosapentaenoic acid) metabolites, respectively. *Turicibater* and *Oscillibater* were positively correlated with nine (5-aminovaleric acid betaine, 4,6-dimitro-o-cresol, Aloin, Chelidonic acid, Guanidinoethyl sulfonate, Imidacloprid, Methyl thiosalicylate, Propenyl thiosulfate, Pumiloside) and two (pentanoic acid, Thr-Gln) metabolites, respectively. In addition, *Oscillibater* was negatively correlated with Maltotetraose. For harmful microbes, positive correlations were observed mainly between the *Lachnospiraceae*_NK4A136_group, *Desulfovibrio* and metabolites Capsaicin, Descladinoseazithromycin, Erucamide, Caulophyllogenin, Maltotetraose and Salvinorin a. In the alum-vs-E515 comparison (Figure 11B), similar metabolite associations were detected for *Turicibacter* and *Staphylococcus* (Methyl thiosalicylate, L-Cysteic acid, L-Cysteinesulfinic acid and Heptadecasphing-4-enine) and Erysipelatoclostridium and Lactobacillus (dicarboxylic acid, Descladinoseazithromycin, Arecoline hydrobromide, D-galacturonic acid, etc.). *Odoribacter* was correlated with four metabolites; three were positively related (phosphocholine, Ala-Phe and Phosphatidylethanolamine) and one was negatively related (Heptadecasphing-4-enine).

### 3.9. Validation of DEGs by RT-qPCR

To verify the results from the RNA-seq analysis, nine DEGs were selected for validation by RT-qPCR. The trends in gene expression obtained from RT-qPCR were similar to those obtained from RNA-seq (Figure S4), thus suggesting that the transcriptomic analysis results were credible.

### 3.10. E515-Bb Immunization Provided Better Protection against Bb Challenge

**Figure 12:**
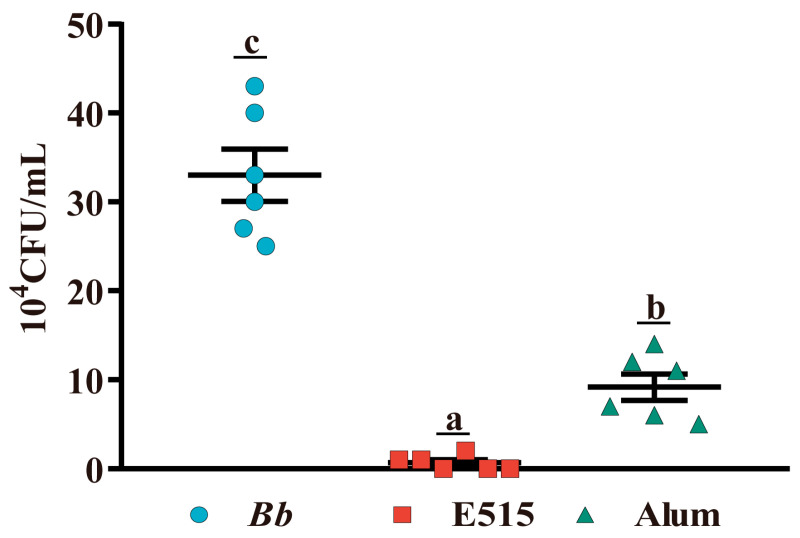
Protective effect of E515-Bb vaccine on mice post-Bb challenge. Mice (n = 9/group) were s.c. injected with 0.2 mL of inactivated Bb antigen (3 × 10^9^ CFU/mL) or Bb antigen adjuvanted with E515 or alum. Then, mice were challenged by an intraperitoneal injection of live Bb (5.2 × 10^9^ CFU/mice). Seven days after attack, bacterial loads in lungs were quantified (n = 6). Data are presented as mean ± SE. Bars with different letters are statistically different (*p* < 0.05).

To evaluate the protective effect of the E515-Bb vaccine, 7 days after the Bb challenge, the bacterial loads in the lungs of mice were investigated. Analysis (Figure 12) showed that compared to the Bb antigen group, the lung bacterial loads in the E515 and alum-adjuvanted Bb vaccine groups were significantly reduced (*p* < 0.05). Furthermore, the bacterial load in the lungs was further reduced in mice treated with the E515-Bb vaccine when compared with the alum-Bb vaccine group (*p* < 0.05).

## 4. Discussion

### 4.1. Humoral Response Induced by E515-Bb Vaccine

Due to the COVID-19 pandemic, there has been a global resurgence of respiratory diseases [19]. With the increasing number of infectious diseases caused by the Bordetella species, including in animals and humans, there is an urgent need to develop novel vaccines to control these respiratory pathogens. In this study, we verified the efficiency of an inactivated Bb vaccine candidate which was formulated with plant oil-based adjuvant E515 and contained α-tocopherol (VE) and ginseng saponins (GS). It found that the inoculation of Bb inactive vaccine adjuvanted with E515 (SO + VE + GS) in mice elicited significantly higher levels of Bb-specific IgG than those containing SO, SO-GS or SO-VE groups, which can be attributed to the orchestrate of them all (Figure 2A). The alum was used as a positive control. Bb-specific-IgG and IgG isotypes can provide good protection from Bb infection [20]. Therefore, we first investigated the humoral immune response induced by Bb vaccine adjuvanted with E515 or alum. We found that compared to the alum (alum-Bb) group, the IgG levels in the E515 (E515-Bb) group were advantageous in the first 3 weeks and peaked 1 week earlier than with the alum (Figure 2B). More specifically, our IgG subtyping results demonstrated the ability of E515 to drive both Th1 and Th2 immune responses with more pronounced Th1-type activity, as indicated by IgG2a priority (Figure 2C). Meanwhile, the addition of sunflower oil with GS as an adjuvant for the NDV vaccine stimulated a specific antibody that peaked 3 weeks after the booster [21]. These distinctions may be related to the different antigens or animals the adjuvant worked with.

### 4.2. Mixed Th1/Th2/Th17 Cellular Responses Induced by E515-Bb Vaccine

TH1, TH2, TH17 and Treg are subtypes of T helper cells; these cells secrete different cytokines and regulate various immune reactions [22]. TH1 type cells are mainly involved in cell-mediated immune responses and are activated by pathogens, including bacteria and viruses. IFN-γ, IgG2a and the transcription factor T-bet are all related to the Th1 immune response. TH2 cells are primarily involved in humoral immune responses, while IgG1, IL-6 and the transcription factor GATA-3 are related to the TH2 immune response [23]. Treg cells play a crucial role in maintaining immune homeostasis by suppressing excessive or inappropriate immune responses. TGF-β1 is also known to be related to the Treg immune response [24]. TH17 cells were discovered relatively recently and are involved in inflammatory responses and host defense against certain bacteria, while IL-17 and the transcription factor ROR-γ are related to the Th17 immune response [25]. Generally, Con A and LPS activate T and B lymphocytes [26]. In the present study, a significant increase in lymphocyte proliferation was observed (Figure 3A) in the expression levels of the transcription factors GATA-3, T-bet and ROR-γt (Figure 3B), Th1 cytokines (IFN-γ), Th2 cytokines (IL-6) and Th17 cytokines (IL-17), along with reduced levels of Treg cytokines (TGF-β1) (Figure 3C–F) in E515- (E515-Bb) immunized groups when compared to the Bb antigen group or alum adjuvant group, thus indicating the induction of a mixed T cell mediated immune response by E515. Previous research verified that the Bordetella colonization factor A (BcfA) elicited protective Th1 and Th17 immune responses [20].

Cytokines and chemokines play crucial roles in regulating immune responses and intercellular communication [27]. Cytokines IL-6, IL-4 and IL-13 are all involved in TH2 cell-mediated inflammation and can effectively stimulate a diverse range of innate and adaptive immune cells, orchestrating critical functions, including immune regulation and antibody production [28,29]. Our current results show that alum- and E515-adjuvanted Bb vaccine groups significantly increased the concentrations of IL-6, IL-4 and IL-13 cytokines (Figure 4A,B). With respect to the adjuvant, previous researchers concurred that eliciting Th1 and Th17 cellular responses is pivotal for generating an effective immune response against Bordetella [1]. The IL-23/IL-17 axis has an essential effect on the host’s defense against bacterial infection [30]. Notably, IL-17 can directly activate immune cells to provide protection against pathogens via an antibody-independent pathway [31]. Chemokine CCL-20/MIP-3α is a newly targeted biomarker due to its promising role in the coordination and modulation of humoral immune responses, particularly memory responses, at the cellular level [32]. Furthermore, CCL-20/MIP-3α not only exhibits antibacterial activity, as a chemokine for immature dendritic cells, effector/memory T cells and B cells, but also exerts strong chemotactic effect on lymphocytes. Furthermore, MIP-3α can recruit helper T cells (Th17) and regulatory T cells (Treg) that produce pro-inflammatory IL17 to the site of inflammation [33]. In our study, cytokine array results further confirmed the elevation of Th1 and Th17 cell-associated cytokines in the E515- (E515-Bb) immunized group, as compared to the alum or Bb antigen groups, including IFN-γ, IL-17, IL-17F, IL-21, IL-22 and IL-23 and chemokine MIP-3α (Figure 4). These findings provided additional evidence that the mice in the E515 group produced enhanced T cell-mediated immune responses.

CD4 and CD8 T cells are two crucial subtypes of T lymphocytes. The higher ratio of CD4+/CD8+ often serves as an indicator of a more robust immune response [34]. In our research, the E515 (E515-Bb) group showed a significantly increased percentage of CD4+ T cells and a ratio of CD4+/CD8+ in splenocytes when compared to the alum (alum-Bb) and Bb antigen groups (Figure 5A). Similar results were obtained when Ginsenoside Rg1 was used as an adjuvant for the porcine reproductive and respiratory syndrome virus (PRRSV) vaccine [35]. Effector and memory CD4+ T cells offer superior B cell assistance, facilitating accelerated B cell proliferation, swift class switching, enhanced antibody production and provide critical contributions to protection against microbial pathogens [36]. Our results indicated that E515 promoted a higher percentage of CD4+ TM and TE cells, while alum induced a high number of CD8+ TM and TE cells (Figure 5B,C). This suggested that E515 and alum exhibit different behaviors in terms of their adjuvant effects on the T cell-mediated immune response to the Bb antigen. Next, we investigated B cell differentiation by flow cytometry. Plasmablasts, are the rapidly generated and ephemeral effector cells in the early antibody response, while plasma cells act as the long-lasting mediators of enduring humoral immunity [37]. Germinal center (GC) affinity-matured B cells are responsible for infection and immunization [38]. As illustrated in Figure 5D–F, there was a notable increase in the numbers of plasmablasts, plasma cells and GC B cells in the E515 group than in the other two groups. This finding further validates the results of the higher level of Bb-specific antibody elicited by the E515-Bb vaccine.

### 4.3. Mechanisms Unraveled by RNA-Seq, 16S rRNA and Metabolomics

RNA-seq-based transcriptomics is one of the most commonly used sequencing tools for uncovering the immune mechanisms of vaccines from the mRNA perspective [39,40,41]. The gut microbiota plays a pivotal role in shaping a host’s immune response and its functionality has been suggested as a potential modulator influencing the response to vaccination [42]. Immunogenicity to vaccines has also been correlated with the gut metabolome [43]. In the present study, transcriptomics (spleen), microbiota sequencing and metabolomics (cecal content) were adopted to investigate the mechanisms of E515 adjuvant working with Bb vaccines. In the E515 group, GO enrichment analysis showed that DEGs were concentrated in the response to stimuli, defense responses and the regulation of immune system processes (Figure S2), thus suggesting an activated immune response to exogenous stimuli. A pathway analysis showed that cytokine–cytokine receptor interaction and the IL-17 signaling pathway were significantly enriched in the two adjuvant groups when compared to the Bb antigen group (Figure 6); this concurred with the identified pathways and the higher levels of cytokines in the serum when stimulated by E515 and alum (Figure 4E). In addition, the chemokine signaling pathway activated by E515 may explain the enhanced production of chemokine MIP-3α by the E515-Bb vaccine (Figure 4B,C).

To gain further insight into the potential links between the gut microbiota and microbial metabolites with the host immune response, 16S rRNA sequencing and the untargeted metabolomics analysis of the cecal contents in the mice were performed. We observed that the structure of the gut microbiota in the E515 group was significantly different from that in the other two groups (Figure 7B,H,I). The mice in the E515 group showed significant reductions in the abundance of Desulfovibrio and the Lachnospiraceae_NK4A136-group and increased the abundance of Alistipes, Odoribacter and Colidextribacter (Figure 7L and Figure 8). Furthermore, the metabolism pathways associated with the “energy, cofactors and vitamins”, “Cell Growth and Death” and “Cell Motility” pathways were obviously enriched in the E515 group (Figure 9). Desulfovibrio can generate LPS to induce inflammation [44] while the Lachnospiraceae NK4A136-group exhibits a discriminative feature of gut dysbiosis [45]. Wang et al. previously demonstrated that resveratrol supplementation restored the gut barrier by reducing the abundance of Desulfovibrio and the Lachnospiraceae_NK4A136_group [46]. Alloprevotella and Odoribacter can synthesize short-chain fatty acids (SCFAs) which are known to benefit health [47] while Colidextribacter is an anti-inflammatory probiotic bacteria that can also produce SCFAs [48,49]. Therefore, it can be inferred that the E515-Bb vaccine enhanced the host’s immune response to maintain a healthy balance of harmful (Desulfovibrio and Lachnospiraceae_NK4A136-group) and beneficial (Alistipes, Odoribacter and Colidextribacter) microbiota in the gut. In turn, these microbiotas can influence cell activity and cell signaling pathways by regulating energy, cofactors and the metabolism of metabolism. It was also observed that the alum mainly increased Lactobacillus and activated pathways, including nucleotide metabolism, glycan biosynthesis and metabolism, thus suggesting that they exerted adjuvant effects by influencing different microbial communities.

Microbial metabolites can access and interact with host cells to influence immune responses. Lipoic acid serves as a vital cofactor in mitochondrial metabolism and is required for cell growth. Lipoic acid is also involved in a number of cellular actions, as an antioxidant and as a mediator of cell signaling pathways [50]. Arachidonic acid (AA) is crucial for tissue homeostasis and health [51]. Previous studies have shown that the toxic effects of kresoxim–methyl (KM) on adult zebrafish are due to disturbances in AA metabolism [52]. Unsaturated fatty acids (UFAs) are integral components of membrane phospholipids and play a critical role in regulating cellular function by virtue of their effects on membrane properties. Previous research implied that IFN-α2b can act against microbes by promoting the biosynthesis of UFAs [53]. In the present study, we found that DEMs in the E515 group were obviously enriched in the metabolism of cofactors and vitamins (Lipoic acid), carbohydrate metabolism (starch and sucrose metabolism), the metabolism of other amino acids (beta-alanine metabolism, taurine and hypotaurine metabolism) and lipid metabolism (arachidonic acid metabolism, primary bile acid biosynthesis, biosynthesis of unsaturated fatty acids) when compared to the alum and Bb vaccine groups (Figure 10E,F). These results suggested that the E515 adjuvant influenced the immune regulatory effects of the Bb vaccines by regulating their energy and lipid metabolism. This finding was also confirmed by our microbiota data. In addition, strong associations between the cecal microbiota and metabolites were detected by Pearson’s correlation coefficient analysis. In the E515 group, metabolites such as 5-aminovaleric acid betaine, 4,6-dimitro-o-cresol and Aloin and Chelidonic acid were positively correlated with beneficial microbes (Colidextribacter, Odoribacter, Turicibater and Oscilli-bater) but negatively correlated with harmful microbes (Lachnospiraceae_NK4A136_group, Desulfovibrio) (Figure 11).

Our study has several limitations that need to be considered. We uncovered the potential role of E515 adjuvant in promoting the immune response to vaccination. Beyond the humoral and cellular immune responses and the multi-omics analysis that have been performed in this study, further investigation is now required to identify the key genes, bacteria strains or metabolites that are activated by the E515-Bb vaccine. Addressing these key issues will provide a better understanding of the effect of plant oil original adjuvant E515.

## 5. Conclusions

In this study, soybean oil, vitamin E and *ginseng* saponins exhibited synergistic adjuvant effects (plant oil-based adjuvant E515) on the immune responses of *Bordetella bronchiseptica* vaccines. The mice in the *Bordetella bronchiseptica* vaccine that was adjuvanted with plant oil showed significantly earlier and higher bacterial-specific antibody responses, strongly enhanced Th1/Th2/Th17 cellular responses and provided better protection from lethal challenge. The potential mechanism may relate to the cytokine–cytokine receptor interaction, the IL-17 signaling pathway and the chemokine signaling pathway, along with the abundances of beneficial bacteria (*Alistipes*, *Odoribacter* and *Colidextribacter*) and the modulation of energy and lipid metabolism. Collectively, the findings suggest the E515 as a promising adjuvant for *Bordetella bronchiseptica* vaccines. As such, the effectiveness of plant oil-based adjuvant E515 in more vaccines in the veterinary field is worthy of investigation.

## Figures and Tables

**Table 1:** Experiment 1—groups and immunization.

Group No.	Mice No.	Vaccine Component	Bb
1	6	Saline	-
2	6	Saline-Bb	3 × 10^9^ CFU/mL
3	6	SO-Bb	3 × 10^9^ CFU/mL
4	6	SO-Ve-Bb	3 × 10^9^ CFU/mL
5	6	SO-Gs-Bb	3 × 10^9^ CFU/mL
6	6	E515-Bb	3 × 10^9^ CFU/mL

**Table 2:** Experiment 2—groups and immunization.

Group No.	Mice No.	Vaccine Component	Bb
1	15	Saline-Bb	3 × 10^9^ CFU/mL
2	15	E515-Bb	3 × 10^9^ CFU/mL
3	15	ISA206-Bb	3 × 10^9^ CFU/mL

**Table 3:** Experiment 3—groups and immunization.

Group No.	Mice No.	Vaccine Componen	Bb
1	9	Saline-Bb	3 × 10^9^ CFU/mL
2	9	E515-Bb	3 × 10^9^ CFU/mL
3	9	ISA206-Bb	3 × 10^9^ CFU/mL

## Data Availability

The data is available from the corresponding author upon request.

## Supplementary Materials

The following are available online at https://www.mdpi.com/article/10.3390/vaccines12101182/s1. Figure S1: the numbers of differentially expressed genes (DEGs). (A) The circle shows the numbers of upregulated and downregulated genes in the three groups. (B) The dynamic Venn diagram shows the DEGs in each comparison group. (C–E) The radar chart shows the top 30 DEGs (with a maximum log2 (fc)) in each of the comparison groups; Figure S2: the GO functional enrichment analysis. (A) Bb-vs-alum, (B) Bb-vs-E515 and (C) alum-vs-E515. The *x*-axis represents the gene percent. The *y*-axis represents the enrichment of the GO terms. The color represents the corrected Q value; Figure S3: the relative abundance (%). (A) The phylum, (B) family and (C) genus; Figure S4: validation of DEGs by RT-qPCR. The log2(Fold change) of the relative mRNA expression levels by RNA-seq and RT-qPCR. Table S1: the sequences of primers for quantitative RT-qPCR.
