# Supplementary material for: Mixed Th1/Th2/Th17 Responses Induced by Plant Oil Adjuvant-Based B. bronchiseptica Vaccine in Mice, with Mechanisms Unraveled by RNA-Seq, 16S rRNA and Metabolomics
Source: Vaccines (Basel). 2024 Oct 17;12(10):1182. doi: 10.3390/vaccines12101182 (PMC11512391; doi:10.3390/vaccines12101182)
Supplement: Supplementary file 1 [file vaccines-12-01182-s001.zip › vaccines-3218665-Supplementary figures.pdf]

Supplementary Data

Mixed Th1/Th2/Th17 responses induced by a plant oil adjuvant-based *Bordetella bronchiseptica* vaccine in mice, and potential underlying mechanisms, as revealed by RNA-seq, 16S rRNA and non-targeted metabolomics analyses

Xuemei Cui [a], Qiuju Xiang [a,b], Yee Huang [a], Quanan Ji [a], Zizhe Hu [a], Tuanyuan Shi [a], Guolian Bao [a,*], Yan Liu [a,*]

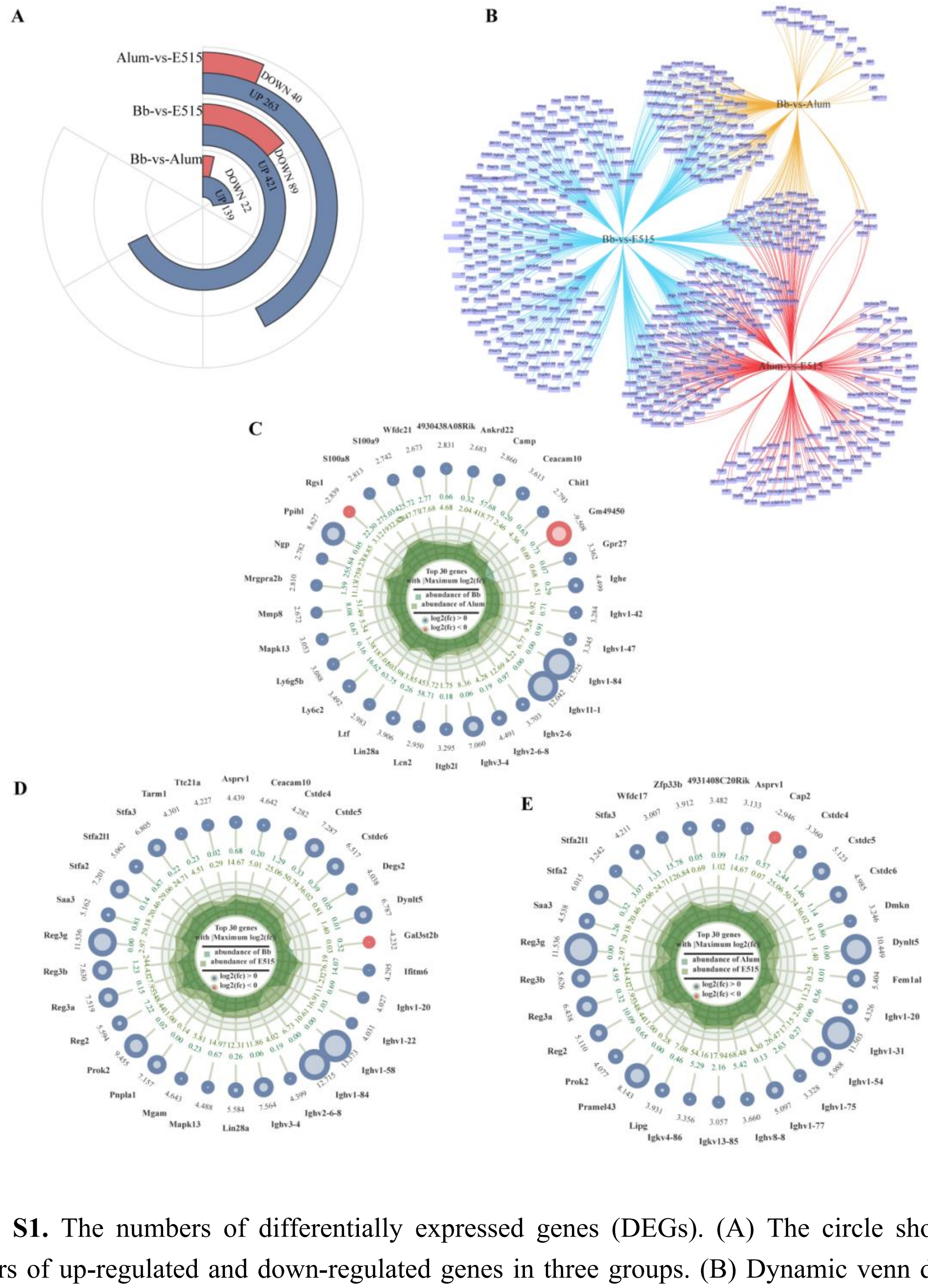

Figure S1. The numbers of differentially expressed genes (DEGs). (A) The circle shows the numbers of up-regulated and down-regulated genes in three groups. (B) Dynamic venn diagram shows DEGs in each comparison groups. (C-E) Radar chart shows the top 30 DEGs (with a maximum log2 (fc)) in each comparison groups.

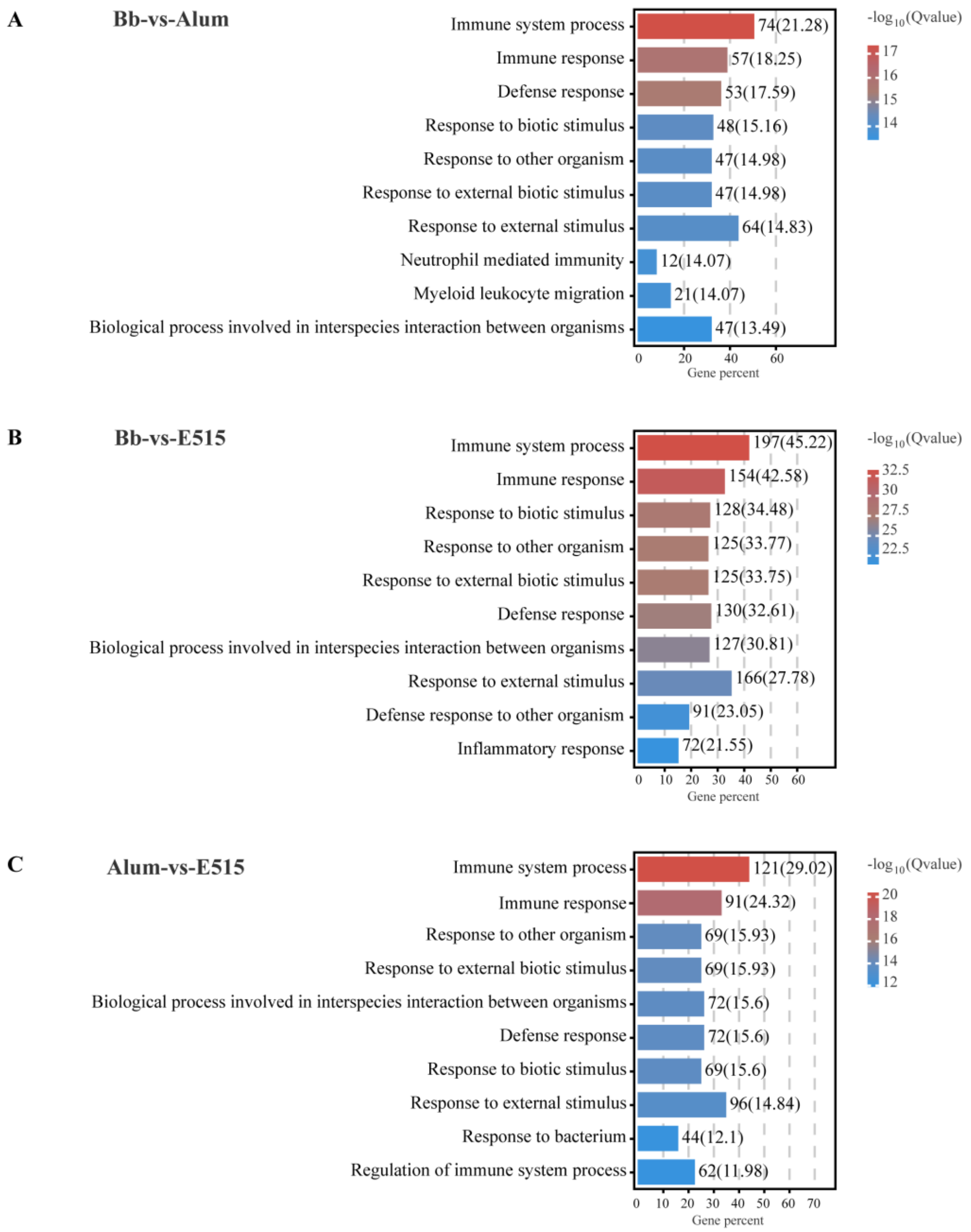

Figure S2. GO functional enrichment analysis. (A) Bb-vs-Alum, (B) Bb-vs-E515, and (C) Alum-vs-E515. The x-axis represents the gene percent. The y-axis represents the enrichment of GO terms. The color represents corrected Q value.

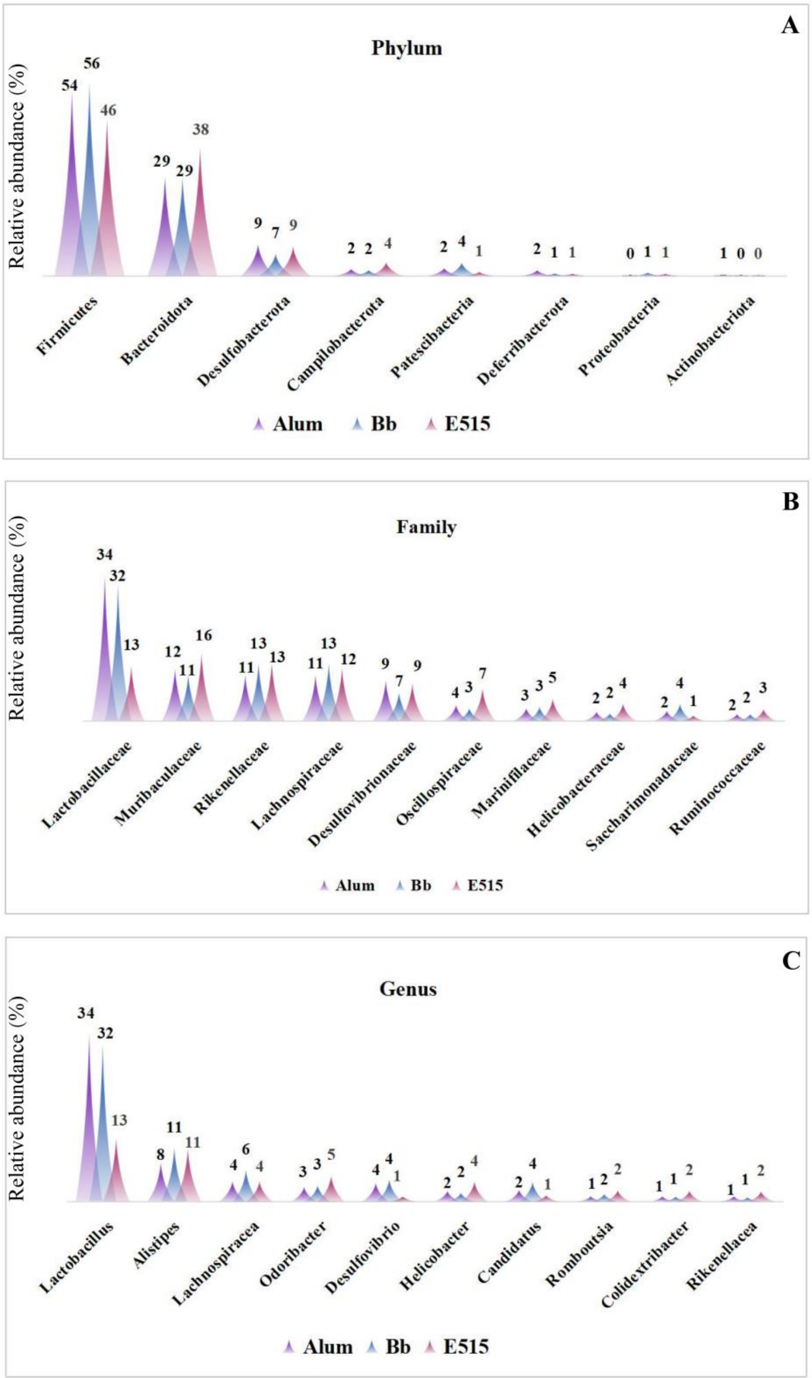

Figure S3. Relative abundance (%). (A) Phylum, (B) Family, and (C) Genus.

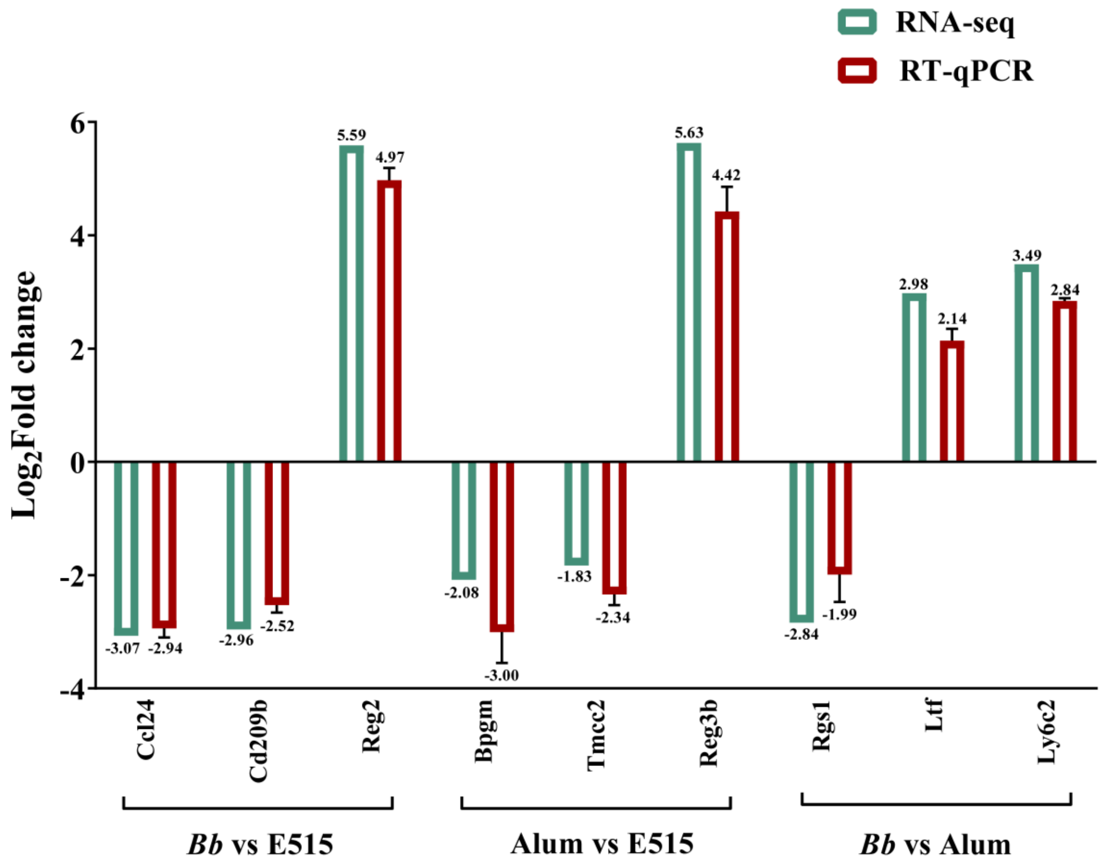

Figure S4. Validation of DEGs by RT-qPCR. Log_2(Fold change) of relative mRNA expression levels by RNA-seq and RT-qPCR .
