# Supplementary material for: Mixed Th1/Th2/Th17 Responses Induced by Plant Oil Adjuvant-Based B. bronchiseptica Vaccine in Mice, with Mechanisms Unraveled by RNA-Seq, 16S rRNA and Metabolomics
Source: Vaccines (Basel). 2024 Oct 17;12(10):1182. doi: 10.3390/vaccines12101182 (PMC11512391; doi:10.3390/vaccines12101182)
Supplement: Supplementary file 1 [file vaccines-12-01182-s001.zip › vaccines-3218665-Supplementary Table.pdf]

Table S1. Sequences of primers for quantitative RT-qPCR

Gene	Primer sequence
(a) GATA-3	Forward: 5-GAG GTG GTG TCT GCA TTC CAA-3
	Reverse: 5-TTT CAC AGC ACT AGA GAC CCT GTTA-3
(b) T-bet	Forward: 5-GTT CCC ATT CCT GTC CTTC-3
	Reverse: 5-CCT TGT TGT TGG TGA GCTT-3
(c) ROR-γt	Forward: 5-CAG TAT GTG GTG GAG TTT GC-3
	Reverse: 5-GCT TCC ATT GCT CCT GCT TT-3
(d) GAPDH	Forward: 5-TCG TCC GGT AGA CAA AAT GG-3
	Reverse: 5-GAG GTC AAT GAA GGG GTC GT-3

Sequences of primers for quantitative RT-qPCR (Validation of DEGs)

Gene	Primer sequence	
(e) Ccl24	Forward: 5-CCT GAA CTT GGA CAT AGG GGA-3	
	Reverse: 5-GCC TTT GAG CCA CAC AAG AG-3	
(f) Cd209b	Forward: 5-TTG GCA GTC TCC AAA ACC CC-3	**Bb vs E515**
	Reverse: 5-GGG ATC CTG GAC GTA AGC TC-3	
(g) Reg2	Forward: 5-TGA AGA CCG TTT GAC CTG GG-3	
	Reverse: 5-GTG CCA ACG ACG GTT ACT TT-3	
(i) Bpgm	Forward: 5-CAT GGT GAA GAG CAG GTA TCTC-3	
	Reverse: 5-GAA CTG GTG AGG CCC AACG-3	
(j) Tmcc2	Forward: 5-TCC TAA GGC TCC CCC TCT TC-3	**Alum vs E515**
	Reverse: 5-TAT CTT GGG GAG AGA CCG CA-3	
(h) Reg3b	Forward: 5-CCT GGT TTG ATG CAG AAC TGGC-3	
	Reverse: 5-ATG GAG CCC AAT CCA AGT GT-3	
(k) Rgs1	Forward: 5-AAC TCC TTG CCA ACC AGA CAGG-3	
	Reverse: 5-TAG TCC TCA CAA GCC AAC CAGA-3	
(l) Ltf	Forward: 5-AGG AAG CAC GGT ATT TGA GGA-3	**Bb vs Alum**
	Reverse: 5-TTG TCA TTC GTG CTT CGG GA-3	
(m) Ly6c2	Forward: 5-CTG TTC CTG AAA CCT GCC CTTC-3	
	Reverse: 5-TGG CAC TCC ATA GCA CTC GT-3	
